# PPAR-*α* Agonist Fenofibrate Decreased Serum Irisin Levels in Type 2 Diabetes Patients with Hypertriglyceridemia

**DOI:** 10.1155/2015/924131

**Published:** 2015-11-26

**Authors:** Xiaomeng Feng, Xia Gao, Yumei Jia, Heng Zhang, Qingrong Pan, Zhi Yao, Ning Yang, Jia Liu, Yuan Xu, Guang Wang, Xinchun Yang

**Affiliations:** ^1^Department of Endocrinology, Beijing Chao-Yang Hospital, Capital Medical University, Beijing 100020, China; ^2^Department of Cardiology, Beijing Chao-Yang Hospital, Capital Medical University, Beijing 100020, China

## Abstract

Irisin is related to insulin resistance and metabolic disorders. The physiologic effects of irisin are partially mediated through peroxisome proliferator-activated receptor-*α* (PPAR-*α*). We investigated the effect of fenofibrate, a PPAR-*α* agonist, on serum irisin in type 2 diabetes patients with hypertriglyceridemia. This study evaluated cross-sectional and interventional studies of 25 type 2 diabetes patients with hypertriglyceridemia (group A) and 40 controls (group B). Group A was treated with fenofibrate (200 mg/day) for 8 weeks. Serum irisin and clinical characteristics were examined. Serum irisin was significantly higher in group A compared with group B (45.15 ± 10.48 versus 35.38 ± 9.97 ng/ml, *P* < 0.001) and correlated with body mass index (*r* = 0.314, *P* = 0.011), fasting blood glucose (*r* = 0.399, *P* = 0.001), total cholesterol (*r* = 0.256, *P* = 0.040), and high-density lipoprotein cholesterol (*r* = 0.247, *P* = 0.047). In multiple regression analysis after controlling for confounders, only fasting blood glucose (*β* = 5.615, *P* < 0.001) and high-density lipoprotein cholesterol (*β* = 19.483, *P* < 0.001) were independently related to serum irisin. After 8 weeks of fenofibrate treatment, serum irisin significantly decreased in group A compared with baseline (45.15 ± 10.48 versus 38.74 ± 12.54 ng/ml, *P* = 0.011). Conclusively, fenofibrate decreased serum irisin in type 2 diabetes patients with hypertriglyceridemia, indicating that PPAR-*α* agonists may protect against metabolic disorders by improving irisin resistance.

## 1. Introduction

The prevalence of type 2 diabetes mellitus (T2DM) is rapidly increasing, and many patients suffer from diabetes-related cardiovascular complications, which are the major cause of death in patients with T2DM. Efforts have been made to reduce the risk of cardiovascular complications, and many previous clinical trials have demonstrated meaningful reduction in the incidence of cardiovascular disorders in T2DM patients after multifactorial risk factor modifications [1, 2].

The dyslipidemia in T2DM is characterized by increased low-density lipoprotein cholesterol (LDL-C), elevated triglycerides (TG), and decreased high-density lipoprotein cholesterol (HDL-C), and it is associated with an increased risk of coronary artery disease [3]. Statins, which decrease LDL-C levels, have been conclusively proven to significantly reduce cardiovascular events in many high-risk patients [4]. However, the residual risk remains after patients have achieved their target LDL-C levels through statins treatment [3]. Combined with statins, fenofibrate has been shown to have highly beneficial effects on lipid metabolism in patients with type 2 diabetes associated with dyslipidemia [5].

Fenofibrate is known as an important peroxisome proliferator-activated receptor-*α* (PPAR-*α*) agonist. Recent data have shown that PPAR-*α* is highly expressed in the liver, kidney, skeletal muscle, endothelium, and vascular smooth muscle [6]. PPAR-*α* agonists are effective at decreasing TG levels, increasing HDL-C levels, changing LDL particle morphology, and playing a pivotal role in regulating insulin resistance, fatty acid oxidation, cellular differentiation, and immune responses, such as inflammation or vascularization related to diabetic complications [7]. Thus, PPAR-*α* agonists are believed to reduce cardiovascular morbidity and mortality [8], independent of their effects on lipid metabolism [9, 10]. Our previous studies have demonstrated that fenofibrate improved vascular endothelial function through comprehensive mechanisms [11, 12]. Although fenofibrate has been proven to reduce diabetic cardiovascular complications [9], its underlying mechanisms are still unknown.

Irisin is a newly identified hormone secreted by myocytes. It reportedly mediates the beneficial effects of exercise and influences multiple metabolic pathways, such as lipid and glucose metabolism [13]. It has been demonstrated that irisin is associated with the development of vascular endothelial function and atherosclerosis [14, 15] and that it is also related to acute coronary syndrome [16–18]. Irisin administration has been proposed as a potential therapeutic tool to treat obesity and diabetes [19]; thus, it may have implications for decreasing cardiovascular risks.

Recent studies have demonstrated that the physiologic effects of irisin are, at least in part, mediated through PPAR-*α* [13]. However, to the best of our knowledge, the effect of fenofibrate on irisin in humans has not been reported. Therefore, in the present study, we aimed to examine whether fenofibrate affected circulating irisin levels in T2DM patients with hypertriglyceridemia.

## 2. Materials and Methods

### 2.1. Subjects

All participants (both genders) ranging in age from 30 to 70 years were recruited from September 2013 to January 2014.

Twenty-five type 2 diabetes mellitus patients with hypertriglyceridemia (group A) were recruited for this study from a group of outpatients at the Department of Endocrinology, Beijing Chao-Yang Hospital, Capital Medical University, Beijing, China. Patients diagnosed with type 2 diabetes mellitus, as defined by the World Health Organization (WHO) criteria, and with stable hypoglycemic treatment for at least 3 months, fasting blood glucose (FBG) levels < 9 mmol/L and glycosylated hemoglobin (HbA1c) levels < 8%, were eligible for the study. Additionally, the patients had been treated with 20 mg/day atorvastatin for more than 3 months; however, their TG levels were still greater than 1.7 mmol/L. The following exclusion criteria for group A were applied: known type 1 and other specific types of diabetes (e.g., genetic defects of the *β*-cell, genetic defects in insulin action, diseases of the exocrine pancreas, endocrinopathies, drug- or chemical-induced diabetes, infections, uncommon forms of immune-mediated diabetes, or other genetic syndromes associated with diabetes) according to the WHO classification of diabetes mellitus, genetic conditions affecting lipids metabolism (e.g., familial hypercholesterolemia and lipoprotein lipase deficiency), changes in hypoglycemic drugs or lipid-lowering drugs during the 3 months preceding the screening visit, any acute cardiovascular event within the last 3 months, and contraindicating treatment with fenofibrate.

Forty healthy people (group B) were recruited as the control group from the community or from the group of people undergoing routine medical check-ups. None of them had a history of prediabetes (including impaired glucose tolerance and impaired fasting glucose), diabetes, hyperlipidemia, or cardiovascular disease.

Moreover, people with hypertension, endocrine disease, systemic inflammatory disease, infectious disease, cancer, chronic kidney disease (i.e., serum creatinine [CR] > 120 *μ*mol/L), hepatic enzymes (i.e., aspartate aminotransferase [AST] and alanine aminotransferase [ALT]) > 1.5 times the upper normal limits, creatine kinase (CK) > 1.5 times the upper normal limit, a history of alcohol abuse, pregnancy, and lactation were also excluded from both groups.

### 2.2. Study Design

Participants in group A were required to attend 3 study visits: the screening visit, visit 1, and visit 2 (spaced 8 weeks apart), while participants in group B attended the screening visit. Starting at visit 1, the group A participants who fulfilled the inclusion criteria (without any exclusion criteria) were administered fenofibrate 200 mg/day for 8 weeks. The capsules were counted at visit 2, and compliance was considered to be satisfactory if > 90% of capsules were taken.

Blood samples and the data on the medical history, height, weight, and blood pressure were collected at the screening visit (groups A and B) and at visit 2 (group A) (under fasting conditions, as described below). At visit 1, each participant in group A received instructions to maintain his/her usual nutritional and exercise habits and to not modify any drug treatment throughout the study. Participants in group A were asked to immediately report the development of unusual muscle soreness or pain throughout the study. In addition, any adverse event in each group A participant was recorded at visit 2.

The study protocol was approved by the Medicine and Pharmacy Ethics Committee of Beijing Chao-Yang Hospital, Capital Medical University. Written informed consent was obtained from each participant prior to the performance of any study procedure.

### 2.3. Data Collection and Laboratory Tests

A complete medical history, including duration and treatment of any disease, was obtained for each participant; height and weight were determined using a standardized protocol. Body mass index (BMI) was calculated as weight (kg)/[height (m)]^2^. Blood pressure was measured using a calibrated standard mercury sphygmomanometer. All readings were measured after a 5 min rest, with the patients in the sitting position.

Fasting blood samples were collected in the morning after an 8-hour overnight fast. FBG, total cholesterol (TC), HDL-C, TG, CR, AST, ALT, CK, and HbA1c were measured in the central laboratory of Beijing Chao-Yang Hospital, Capital Medical University. LDL-C was calculated using the Friedewald formula (LDL = CHOL − [TG/5 + HDL]). Serum samples from all participants were stored at −80°C. Serum irisin concentrations were measured in duplicate at the same time using enzyme-linked immunosorbent assay (ELISA) kits (Phoenix Pharmaceuticals Science, Inc., USA) for quantitative detection with an automated ELISA reader (VARIOSKAN FLASH-5250040, Thermo Scientific, USA).

Adverse events were recorded throughout the study. The safety parameters included serum CR, AST, ALT, and CK.

### 2.4. Statistical Analysis

All analyses were performed with Statistical Package for Social Sciences version 19.0 (SPSS, Inc., Chicago, IL, USA). Data were expressed as the mean ± SD. Comparisons of the baseline clinical and biochemical markers, as well as the irisin levels, between groups A and B were performed using an independent sample *t*-test. Comparisons of the pretreatment and posttreatment (with fenofibrate) clinical and biochemical markers, as well as the irisin levels, in group A were performed with a paired *t*-test. Proportions were analyzed using the chi-squared test. The association between the baseline values of irisin and the other baseline parameters was examined using Pearson's and Spearman's correlation coefficient analyses and multiple stepwise regression analysis. In all statistical tests, *P* values < 0.05 were considered to be significant, and all tests were two-sided.

## 3. Results

### 3.1. Baseline Clinical Characteristics of the Study Participants

The baseline clinical characteristics of the study participants are listed in Table 1. The participants in groups A and B were similar in sex, age, systolic blood pressure (SBP) and diastolic blood pressure (DBP) levels, and TC levels (*P* > 0.05 for all). The FBG (*P* < 0.001), LDL-C (*P* = 0.023), TG (*P* < 0.001), and HbA1c (*P* < 0.001) levels were higher, and the HDL-C levels (*P* < 0.001) were lower in group A compared with group B.

### 3.2. Baseline Serum Irisin Levels of the Study Participants

**Figure 1:**
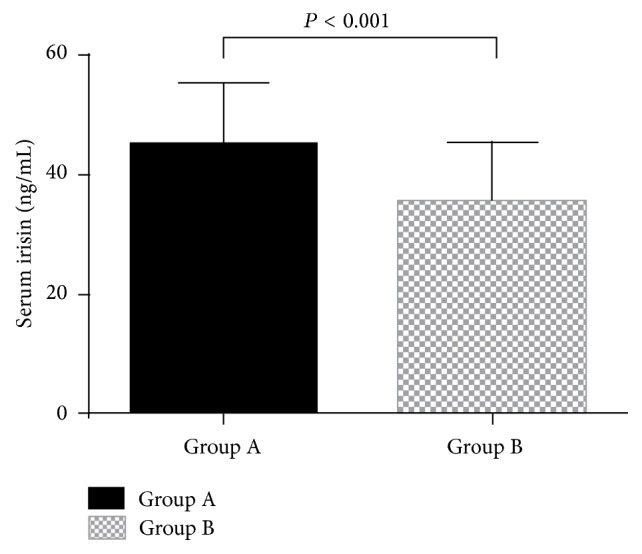
Baseline serum irisin levels in the study participants. The values are expressed as the means ± SD. Group A: type 2 diabetes patients with hypertriglyceridemia (*n* = 25). Group B: control subjects (*n* = 40).

The fasting serum levels of irisin were significantly higher in group A than in group B (45.15 ± 10.48 versus 35.38 ± 9.97 ng/mL, *P* < 0.001) (Figure 1).

### 3.3. Correlation between Serum Irisin Levels and the Baseline Parameters

The following parameters were found to be significantly correlated with the serum irisin levels: BMI (*r* = 0.314, *P* = 0.011), FBG (*r* = 0.399, *P* = 0.001), TC (*r* = 0.256, *P* = 0.040), and HDL (*r* = 0.247, *P* = 0.047) (Table 2).

Multiple stepwise regression analysis was performed to determine the parameters that were independently associated with serum irisin. The results showed that only FBG (beta coefficient 5.615, SE 0.903, standard beta coefficient 0.691, *P* < 0.001) and HDL (beta coefficient 19.483, SE 3.674, standard beta coefficient 0.598, *P* < 0.001) were independently related to serum irisin levels (Table 2). The multiple regression equation was *Y*_Irisin_ = −23.091 + 5.615*X*_FBG_ + 19.483*X*_HDL_. The model had an adjusted *R* squared of 0.403, *F* = 22.616, and *P* < 0.001.

### 3.4. Effect of Fenofibrate on the Clinical Characteristics in Group A

The pretreatment and posttreatment (with fenofibrate) clinical parameters in group A are summarized in Table 3. Compared with baseline, at visit 2, the patients in group A presented significantly lower levels of TG (*P* < 0.001) but significantly higher levels of HDL-C (*P* < 0.001). In addition, no statistically significant changes were observed in BMI, SBP, DBP, FBG, TC, LDL, AST, ALT, CK, and CR after 8 weeks of fenofibrate treatment compared with baseline (*P* > 0.05 for all).

### 3.5. Effect of Fenofibrate on the Serum Levels of Irisin in Group A

**Figure 2:**
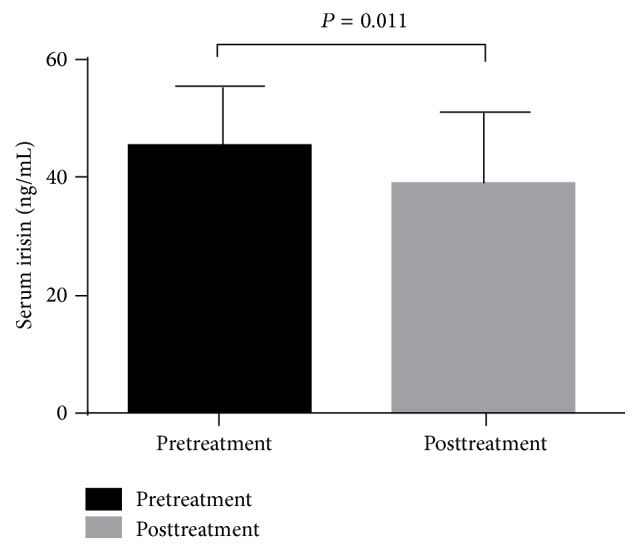
Serum irisin levels in type 2 diabetes patients with hypertriglyceridemia after 8 weeks of fenofibrate treatment compared with the baseline levels. The values are expressed as the means ± SD (*n* = 25).

After 8 weeks of fenofibrate treatment, the serum irisin levels in group A were significantly decreased compared with the baseline levels (from 45.15 ± 10.48 ng/mL at pretreatment to 38.74 ± 12.54 ng/mL after treatment, *P* = 0.011) (Figure 2).

### 3.6. Safety Parameters

All participants completed the study, and no serious adverse effects were observed throughout the study.

## 4. Discussion

Skeletal muscle tissue is an important organ for lipid and glucose metabolism. It secretes cytokines and peptides that are classified as “myokines” [20], which act as endocrine hormones and regulate whole body metabolism. Irisin is a newly identified myokine. Recently, comprehensive animal and human studies have provided convincing evidence of a link between insulin resistance, lipid and glucose metabolism, and irisin. However, the data have been inconsistent.

In this study, we demonstrated that serum irisin levels were significantly higher in the T2DM patients with hypertriglyceridemia compared with the controls, and serum irisin was positively correlated with BMI, FBG, TC, and HDL, suggesting that irisin could play an important role in the delicate balance of energy metabolism and insulin resistance.

Our findings are in contrast to some recent studies indicating that circulating irisin was significantly lower in patients with T2DM [21, 22]. However, our results align with other studies indicating that circulating irisin was significantly higher in patients with insulin resistant diseases, such as metabolic syndrome [23] and polycystic ovary syndrome [24], and that it was associated with an increased risk of metabolic syndrome, cardiometabolic variables, and cardiovascular diseases in humans. There may be a compensatory increase of irisin to overcome insulin resistance [25]. We hypothesize that the increased irisin levels in T2DM with hypertriglyceridemia in our study might have represented irisin resistance, reflecting a compensatory result to counterbalance the increasing needs for irisin (similar to the increased insulin levels in insulin resistance) and to improve metabolic features. Additionally, we also hypothesize that, at different stages of T2DM, irisin levels might change from overcompensating to failing to compensate (similar to different insulin levels in different stages of T2DM). These hypotheses require further study in the future.

Moreover, the reported results on the association between irisin, obesity, and metabolic parameters have been controversial. The majority of previous studies align with our findings that circulating irisin levels were positively associated with FBG levels [21, 23, 26, 27]. While some authors have supported our findings with reports of a positive correlation between circulating irisin levels and BMI [21, 24, 26], others have reported a negative correlation [22]. Furthermore, our findings are consistent with some previous studies supporting that irisin was positively associated with HDL [27], while a negative association has been observed in other studies [28]. It is possible that these conflicting data are caused by the polypharmacotherapy and other confounding variables of the study populations, such as age, sex, race, or the level of physical activity of the subjects. These discrepancies might also be related to differences in the assays used by the different studies, which may provide different results for the irisin levels.

Accumulating evidence has demonstrated that PPAR-*α* is an important modulator of metabolic syndrome and that it might be a therapeutic target for treating some of its features. PPAR-*α* plays a critical role in lipid metabolism. Its known that target genes are involved in most aspects of lipid metabolism and lipid transport [29]. PPAR-*α* agonist fenofibrate has been proven to be effective at improving lipid parameters. In our study, the T2DM patients with hypertriglyceridemia presented significantly lower levels of TG, whereas they demonstrated significantly higher levels of HDL-C after 8 weeks of fenofibrate treatment compared with baseline.

Importantly, we report for the first time here that fenofibrate treatment administered to T2DM patients with hypertriglyceridemia for 8 weeks resulted in a significant decrease in serum irisin levels, although fenofibrate was reported to increase irisin gene expression in diet-induced male obese mice [30]. These inconsistent data may also be attributed to the heterogeneity of the study subjects, such as species, disease, or the level of physical activity of study subjects. Some previous studies have demonstrated that irisin levels are positively associated with insulin resistance [15, 23–25]. T2DM patients with hypertriglyceridemia have insulin resistance and then present higher irisin levels compared with the controls, which might be a compensatory increase (irisin resistance). T2DM patients with hypertriglyceridemia might need more irisin to overcome irisin resistance for improving insulin resistance and metabolic features [25]. Besides regulating lipid metabolism, treatment with PPAR-*α* activators dramatically improved insulin resistance and glycemic control in db/db mice and OLETF rats [31, 32]. Recent studies of PPAR-*α* agonists have supported that patients with insulin resistance benefit from fibrate therapy [9, 33]. Therefore, we speculate that PPAR-*α* agonist fenofibrate could relieve irisin resistance by improving lipid and glucose metabolism and insulin resistance, thereby decreasing the needs for irisin and reducing circulating irisin levels.

In addition, it has been observed that the overexpression of PPARr coactivator-1alpha (PGC-1*α*) in mouse muscles induced the expression of fibronectin type-III domain containing protein 5 (FNDC5). FNDC5 was supposedly cleaved and released as a novel messenger molecule called irisin, which increased the uncoupling protein-1 (UCP1) levels to induce browning of subcutaneous adipocytes and thermogenesis [13]. Gene arrays indicated that the increased expression of UCP1 and browning of white adipocyte tissue (WAT) by FNDC5 were at least partially mediated through PPAR-*α*. FNDC5 induced a threefold increase in PPAR-*α* mRNA levels in white adipocytes differentiated from stromal vascular cells. Moreover, pharmacological inhibition with a PPAR-*α*-selective antagonist limited the induction of the browning programme by FNDC5 [13]. Therefore, we hypothesize that the PPAR-*α* agonist fenofibrate could induce the increase of UCP1 and browning of WAT by activating PPAR-*α* and that the increased UCP1 levels and browning programme might compensatively inhibit the FNDC5 expression and then reduce the irisin levels. The result of decreased serum irisin levels induced by fenofibrate treatment in our study also supports the hypotheses. The mechanism of this phenomenon should be further investigated.

Furthermore, our result that the serum irisin levels were significantly decreased after the 8-week fenofibrate treatment suggests that fenofibrate might protect against cardiovascular diseases, except for a lipid-lowering effect [9, 10].

Atherosclerosis remains a major risk factor for cardiovascular diseases. PPAR-*α* agonists may induce positive effects on atherosclerotic lesions. Fenofibrate decreased atherosclerotic lessons in a mouse model of mixed dyslipidemia [34]. PPAR-*α* agonists exhibited anti-inflammatory effects in vascular cells by inhibiting the production of some inflammatory cytokines [35, 36], increasing the expression of endothelial nitric oxide synthase (eNOS) and the production of nitric oxide (NO) [37], and improving vascular endothelial function. Our previous study demonstrated that fenofibrate improved coronary flow velocity reserve and arterial stiffness in patients with hypertriglyceridemia [11], and it upregulated tetrahydrobiopterin levels by increasing the expression of guanosine triphosphate cyclohydrolase-I in human umbilical vein endothelial cells [12]. Moreover, activation of PPAR-*α* protected the heart from ischemia/reperfusion injury [38, 39]. The Fenofibrate Intervention and Event Lowering in Diabetes (FIELD) study revealed that total cardiovascular events were significantly reduced through fenofibrate treatment [9].

Reduction of serum irisin levels through fenofibrate treatment may partially explain the beneficial effects of PPAR-*α* agonist treatment in clinical trials in which the favorable effects only partly correlated with lipid changes [9]. Recent evidence has indicated that irisin is associated with cardiovascular diseases. Some studies have demonstrated that circulating irisin levels were positively correlated with endothelium dependent arterial dilation [14] and positively associated with carotid artery intima-media thickness in humans [15]. In addition, increased irisin levels were associated with the development of major adverse cardiovascular events (MACE) in patients with established coronary artery disease after percutaneous coronary intervention (PCI) [16]. Furthermore, some studies have shown that irisin levels gradually decreased after acute myocardial infarction [17, 18].

Further study is required to determine whether the protective effects of fenofibrate against the cardiovascular complications of diabetes are related to irisin. Our findings in the present study may provide one possible pathway to protect against the cardiovascular complications of diabetes with fenofibrate treatment.

Many studies have been published with irisin levels changing widely in human serum or plasma measured by commercial ELISA kits from different companies. The ELISA kits used in our study were from Phoenix Pharmaceuticals, and these kits were reported to have high sensitivity and excellent specificity for detection of human irisin with no significant cross-reactivity or interference. Therefore, they have been widely used in human and animal studies. However, a recent study has questioned the existence of circulating human irisin both because human FNDC5 has a noncanonical ATA translation start and because many human irisin antibodies used in commercial ELISA kits are based on polyclonal antibodies (pAbs) which are not previously tested for cross-reacting serum proteins and lack required specificity [40]. Four commercial pAbs were analyzed by western blot in this study, which revealed that prominent cross-reactivity with nonspecific proteins was in human and animal sera and that some of the previous results measured by ELISA might be presented at high levels [40]. Nevertheless, this study had several methodological deficiencies. First, their method was the only incomplete deglycosylation, which failed to detect irisin in human serum at 12 kDa by western blot which relied on deglycosylation by only one enzyme, namely, PNGase F. Second, a method of mass spectrometry was used, which randomly sampled peptides for detection from all the peptides contained in the sample. The method would be suboptimal for detecting irisin because the peptides of irisin could be missed in complex samples due to their low abundance. Third, this study revealed that the lowest detection limit for irisin by western blot was about 100 ng/mL. Although the values of irisin in some reports were more than 100 ng/mL [23, 24], there were still many other reports in which human irisin levels were below 100 ng/mL [18, 41–45], and some of their ELISA kits were also from Phoenix Pharmaceuticals [18, 41]. Moreover, another study has unequivocally demonstrated that human irisin exists and circulates by tandem mass spectrometry [46]. These widely changing data are possibly owing to the complex discrepancies of the study subjects and the assays used by the different studies. It was reported that the values of irisin measured in the same subjects by the ELISA kits from Phoenix Pharmaceuticals were slightly lower than those using the ELISA kits from other companies [23, 24]. Given the possibility of inaccurate high irisin levels measured by the commercial ELISA kits due to the deficiency of specificity in polyclonal antibodies (pAbs), the irisin ELISA kits from Phoenix Pharmaceuticals may be relatively accurate.

The limitations of our study are identified as follows. Firstly, our study population was limited to the Chinese. Therefore, our findings may not be directly applicable to other populations. Secondly, our sample size was relatively small so that our findings were not powerful enough to account for potentially confounding factors in our analysis, and our results could be improperly influenced by some outliers due to the sample size. Thirdly, the insulin levels were not examined in the present study. Thus, the points that circulating irisin levels were positively associated with insulin resistance and that fenofibrate improved insulin resistance were supported by the previous studies rather than our study. Fourthly, the cross-sectional design of the study does not allow us to determine a causal relationship, but it can certainly raise credible hypotheses to be confirmed and extended by future prospective cohort and mechanistic studies. Finally, since commercial ELISA kits of irisin are mostly based on pAbs which relatively lack specificity and are not previously tested for cross-reacting serum proteins, the values of human irisin measured by ELISA might be at high levels [40, 46].

## 5. Conclusions

We reported the novel finding of a significant increase of serum irisin, a novel myokine, in type 2 diabetes mellitus patients with hypertriglyceridemia. More importantly, we presented novel data that fenofibrate treatment significantly decreased serum irisin levels in type 2 diabetes mellitus patients with hypertriglyceridemia. These results indicate that PPAR-*α* agonists may play an important role in protecting against metabolic disorders by improving irisin resistance. The physiologic and pathologic significance of our findings remain to be further elucidated.

## Figures and Tables

**Table 1:** Baseline clinical characteristics of the study participants.

Parameters	Group A (*N* = 25)	Group B (*N* = 40)	*P* value
Sex (M/F)	19/6	28/12	0.599
Age (years)	53.76 ± 8.89	49.10 ± 10.57	0.071
BMI (kg/m^2^)	26.46 ± 4.60	24.63 ± 3.71	0.083
SBP (mmHg)	125.00 ± 7.65	123.63 ± 9.00	0.529
DBP (mmHg)	74.24 ± 8.74	73.75 ± 6.13	0.808
FBG (mmol/L)	7.43 ± 1.01	5.06 ± 0.50	<0.001
TC (mmol/L)	4.62 ± 0.66	4.49 ± 0.74	0.461
HDL (mmol/L)	1.25 ± 0.29	1.61 ± 0.29	<0.001
LDL (mmol/L)	2.80 ± 0.53	2.45 ± 0.62	0.023
TG (mmol/L)	3.05 ± 0.86	0.92 ± 0.37	<0.001
HbA1c (%)	6.88 ± 0.72	5.42 ± 0.33	<0.001

Group A, type 2 diabetes mellitus patients with hypertriglyceridemia; group B, control subjects; BMI, body mass index; SBP, systolic blood pressure; DBP, diastolic blood pressure; FBG, fasting blood glucose; TC, total cholesterol; HDL-C, high-density lipoprotein cholesterol; LDL-C, low-density lipoprotein cholesterol; TG, triglycerides; HbA1c, glycosylated hemoglobin.

**Table 2:** Correlation and multiple regression analyses of the baseline parameters associated with serum irisin levels.

Parameters	Correlation		Multiple regression	
	*r*	*P* value	*β*	*P* value
Age (years)	0.215	0.085		
BMI (kg/m^2^)	0.314	0.011		
SBP (mmHg)	0.126	0.316		
DBP (mmHg)	−0.107	0.395		
FBG (mmol/L)	0.399	0.001	5.615	<0.001
TC (mmol/L)	0.256	0.040		
HDL (mmol/L)	0.247	0.047	19.483	<0.001
LDL (mmol/L)	0.109	0.387		
TG (mmol/L)	0.230	0.065		
HbA1c (%)	0.239	0.055		

BMI, body mass index; SBP, systolic blood pressure; DBP, diastolic blood pressure; FBG, fasting blood glucose; TC, total cholesterol; HDL-C, high-density lipoprotein cholesterol; LDL-C, low-density lipoprotein cholesterol; TG, triglycerides; HbA1c, glycosylated hemoglobin. The following variables were included in the multiple stepwise regression analysis: age, sex, BMI, SBP, DBP, FBG, TC, HDL-C, LDL-C, TG, and HbA1c.

**Table 3:** Pretreatment and posttreatment clinical characteristics of type 2 diabetes patients with hypertriglyceridemia treated with fenofibrate.

Parameters	Pretreatment (*N* = 25)	Posttreatment (*N* = 25)	*P* value
BMI (kg/m^2^)	26.46 ± 4.60	26.37 ± 4.59	0.209
SBP (mmHg)	125.00 ± 7.56	125.48 ± 6.76	0.668
DBP (mmHg)	74.24 ± 8.74	73.92 ± 7.30	0.831
FBG (mmol/L)	7.42 ± 1.01	7.32 ± 1.00	0.215
TC (mmol/L)	4.62 ± 0.66	4.83 ± 0.69	0.145
HDL (mmol/L)	1.25 ± 0.29	1.47 ± 0.27	<0.001
LDL (mmol/L)	2.80 ± 0.53	2.82 ± 0.66	0.845
TG (mmol/L)	3.05 ± 0.86	1.84 ± 0.76	<0.001
AST (U/L)	22.08 ± 7.33	23.84 ± 9.04	0.322
ALT (U/L)	26.00 ± 5.69	24.44 ± 11.64	0.518
CR (*μ*mol/L)	71.92 ± 13.74	76.10 ± 17.29	0.164
CK (U/L)	84.64 ± 27.69	91.20 ± 37.28	0.307

BMI, body mass index; SBP, systolic blood pressure; DBP, diastolic blood pressure; FBG, fasting blood glucose; TC, total cholesterol; HDL-C, high-density lipoprotein cholesterol; LDL-C, low-density lipoprotein cholesterol; TG, triglycerides; AST, aspartate aminotransferase; ALT, alanine aminotransferase; CR, creatinine; CK, creatine kinase.
